# 664. Attitudes about COVID and Community Engagement with Patients (ACCEPt): Global Survey Reveals the Ongoing Impact of Long COVID-19 on Medically Vulnerable Populations, Five Years On

**DOI:** 10.1093/ofid/ofaf695.219

**Published:** 2026-01-11

**Authors:** Roger Paredes, Jennifer Bodie, Nikola Brigden, Antonella Cardone, Ashley S Cha-Silva, Maria Fernandez, Daniel Gallego, Romina Quercia, Jason Resendez, Doris Christiane Schmitt, Frank Spinelli, Mariano Votta

**Affiliations:** Hospital Universitari Germans Trias i Pujol, Badalona, Catalonia, Spain, Badalona, Catalonia, Spain; Pfizer Inc., New York, New York; Forgotten Lives, Hedon, England, United Kingdom; Cancer Patients Europe, Brussels, Brussels Hoofdstedelijk Gewest, Belgium; Pfizer, New York, New York; Pfizer, New York, New York; European Kidney Patients Federation, Aldaya, Comunidad Valenciana, Spain; Pfizer, New York, New York; National Alliance of Caregiving, Washington, Washington; Path Biobank Foundation, Munich, Bayern, Germany; Pfizer Inc., New York, New York; Active Citizenship Network (Cittadinanzattiva), Rome, Lazio, Italy

## Abstract

**Background:**

COVID-19 continues to disproportionately impact medically vulnerable populations. To assess COVID-19’s ongoing burden on these individuals and address data gaps in post-pandemic care, the COVID Community Council (The Council), eight global representatives from medically vulnerable patient organizations, co-created the ‘Attitudes about COVID and Community Engagement with Patients’ (ACCEPt) survey.

**Figure:**
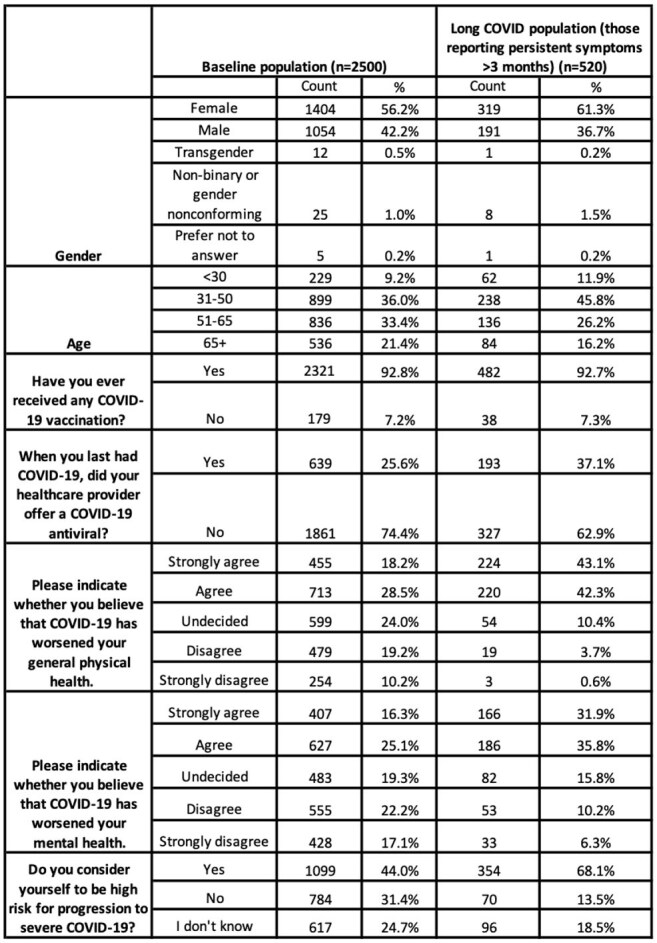
Comparison of survey responses between baseline and Long COVID populations

**Figure:**
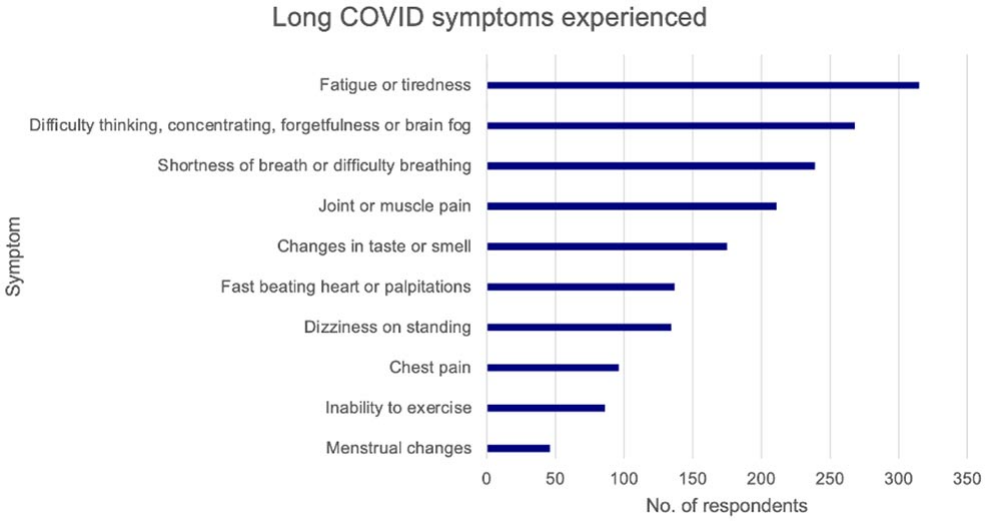
Prevalence of reported Long COVID symptoms among respondents (n=520)

**Methods:**

The survey included adults with risk factors for progression to severe COVID-19 and self-reported COVID-19 diagnosis since January 2022 (irrespective of previous diagnoses) from the United States, Spain, Germany, Australia, and Canada. The 41-question online survey assessed knowledge, attitudes, and practices across several domains, including Long COVID symptoms (defined here as persistent symptoms lasting for >3 months). Self-reported responses were analyzed using descriptive summary statistics.

**Figure:**
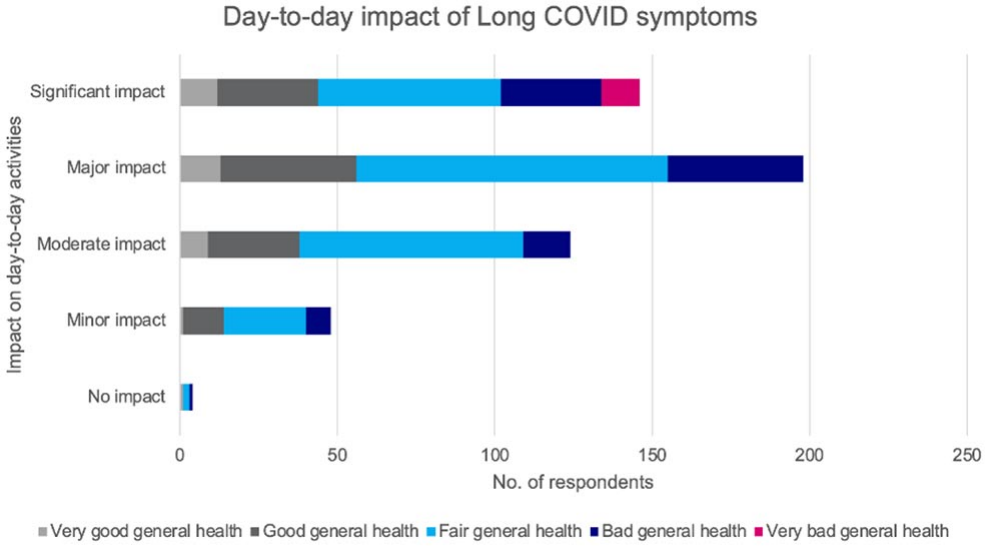
Correlation between Long COVID symptom impact on daily life and self-reported general health (n=520)

**Figure:**
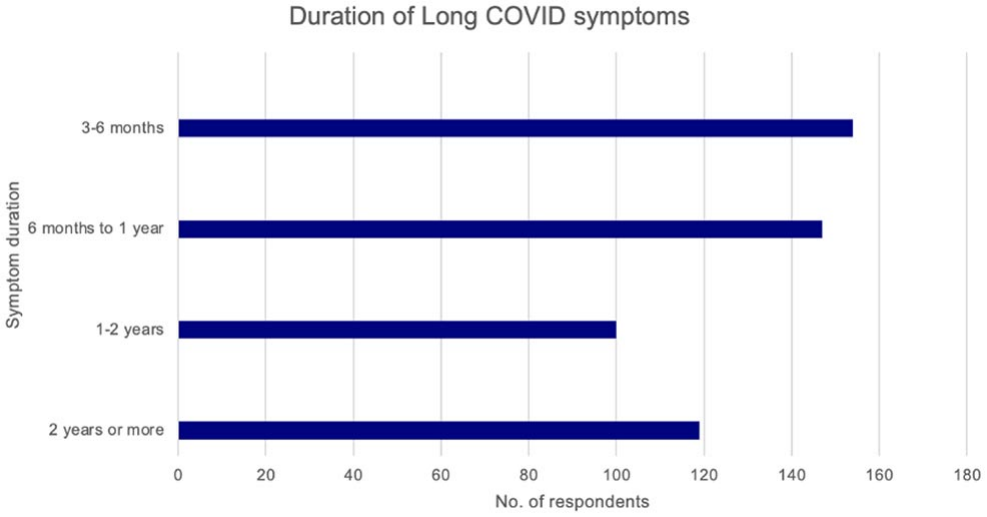
Reported duration of Long COVID symptoms by number of respondents (n=520)

**Results:**

Of 2500 respondents, 20.8% reported Long COVID symptoms, of whom 42.1% endured symptoms >1 year and 22.9% for >2 years. Of those with Long COVID symptoms, most (90.0%) reported moderate to significant impact on daily life, with 85.4% and 67.7% agreeing that COVID-19 had worsened their physical and mental health, respectively. All respondents in “very bad” general health noted a significant daily impact vs 33.3% in "very good" health. While most were vaccinated against COVID-19 (92.7%), 29.3% had not received a vaccine dose in >2 years. Despite meeting survey inclusion criteria for risk factors for severe COVID-19, 31.9% did not consider themselves high-risk. 62.9% were not offered antiviral treatment during their most recent acute COVID-19 illness. Only 48.3% of those with Long COVID symptoms were diagnosed with Long COVID, and many (44.8%) received less than moderate support from friends, family, and caregivers. Further, of the 76.9% who consulted a healthcare provider (HCP), 41.3% noted receiving less than moderate support.

**Conclusion:**

Over 1 in 5 medically vulnerable individuals globally face ongoing effects of COVID-19, often with limited support. Self-reported findings from respondents of this survey highlight the urgent need for improved HCP education and support systems for Long COVID in these populations.

**Disclosures:**

All Authors: No reported disclosures

